# Genetically engineered bacteria and microalgae expressing a mutant of cytochrome P450 BM3 for efficient Diuron degradation in wastewater treatment

**DOI:** 10.1128/spectrum.02905-24

**Published:** 2025-04-16

**Authors:** Christian Helvig, Thamali Kariyawasam, Bas Vriens, Martin Petkovich

**Affiliations:** 1 Department of Biomedical and Molecular Sciences, Queen's University 244870, Kingston, Ontario, Canada; 2 Department of Geological Sciences and Engineering, Queen's University 468059 , Kingston, Ontario, Canada; 3 Beaty Water Research Center, Queen's University 549149, Kingston, Ontario, Canada; Ruhr-Universitat Bochum, Bochum, Germany

**Keywords:** emerging contaminants, wastewater treatment, metabolism, genetically engineered, cytochrome P450, algae, bacteria, pesticides, drug metabolism

## Abstract

The diverse members of the cytochrome P450 superfamily conduct the metabolism of a wide variety of compounds, both endogenous and xenobiotic, thus offering potential for degrading emerging contaminants such as prescription drugs and pesticides in wastewater treatment and aquaculture. This study demonstrates the expression and activity of the mutant P450 enzyme of CYP102A1 (BM3 MT35) engineered in *Bacillus megaterium* and *Chlamydomonas reinhardtii*, harnessed for the degradation of the herbicide Diuron. The P450 BM3 MT35 enzyme was transgenically expressed in *Escherichia coli* and purified via a His6-tag system, with its activity confirmed by spectral and *in vitro* metabolism assays. During *in vivo* experiments, transgenic *Bacillus megaterium* degraded 23% of Diuron after 24 h, reaching 65% by day 5 in Terrific Broth (TB) media. In synthetic and municipal wastewater, Diuron degradation reached 45% and 15%, respectively. No Diuron metabolism occurred in wild-type (WT) *Bacillus megaterium* in all the above tested conditions. Additionally, transgenic *Chlamydomonas reinhardtii* expressing P450 BM3 MT35 in the chloroplast also exhibited enhanced Diuron degradation (52%) compared to WT strains (6%). Overall, our results demonstrate the potential of genetically engineered bacteria and microalgae with cytochrome P450 as a viable strategy for the enhanced treatment of emerging contaminants in wastewater, potentially offering a new, sustainable alternative approach to pollution mitigation.

**IMPORTANCE:**

With a growing number and variety of prescription drugs, pesticides, food additives, and chemicals produced, wastewater is being contaminated with an increasing number of emerging pollutants that cannot be eliminated through classical wastewater treatment. New methods should therefore be developed to remove or deactivate these contaminants. Here, we demonstrate that by using genetically engineered bacteria and microalgae expressing a mutated enzyme, it is possible to efficiently metabolize a targeted pesticide, in this case, Diuron. These new findings should open the door to new ways to treat wastewater by developing low-cost and efficient modified microorganisms that will be able to specifically detoxify past and new emerging water contaminants that cannot be eliminated through classical wastewater treatment.

## INTRODUCTION

**Fig 1:**
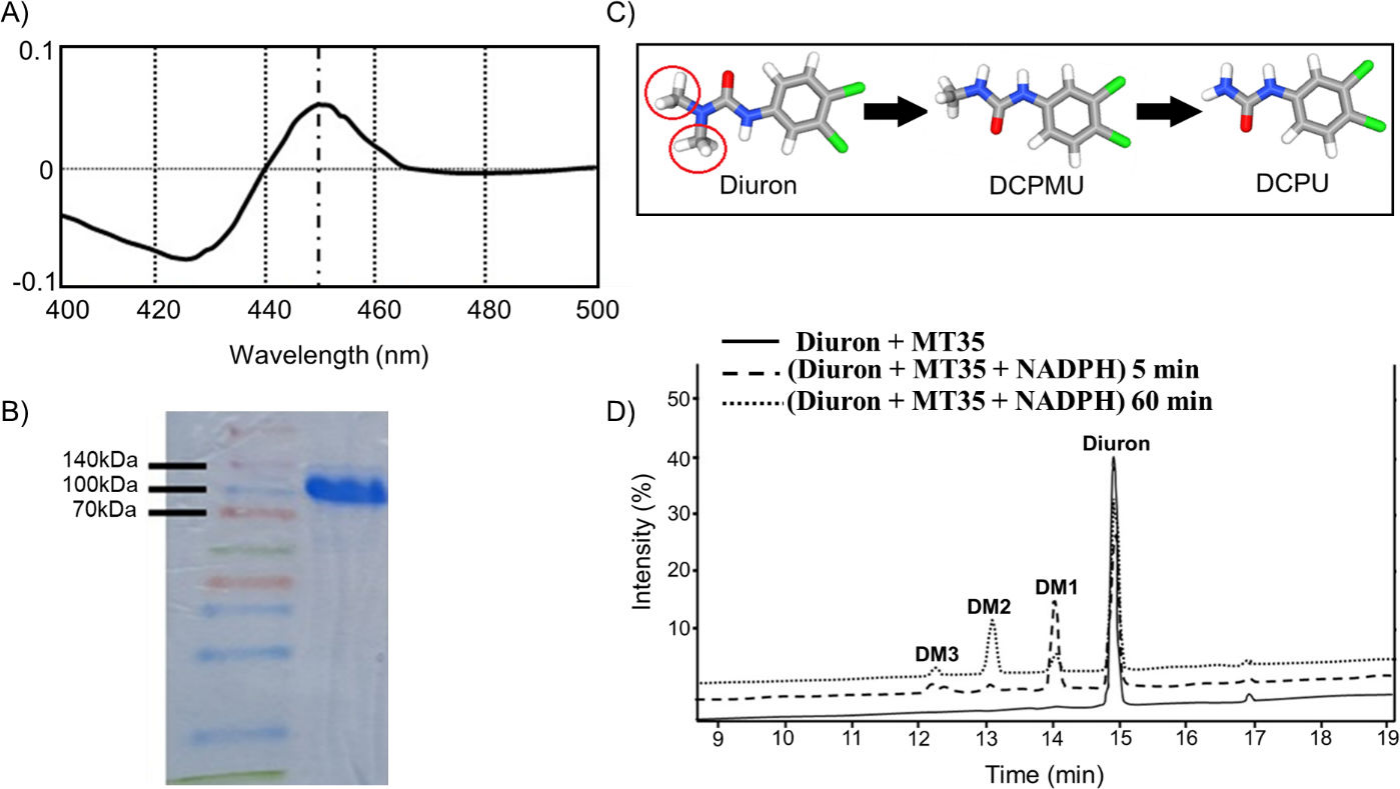
*In vitro* incubation of the purified P450 BM3 MT35 with Diuron. (**A**) Purified P450 BM3 MT35 showed the characteristic P450 peak at 450 nm. (**B**) SDS-PAGE gel image of purified P450 BM3 MT35 on a Ni-NTA column showing a band at 118 kDa characteristic of P450 BM3 protein. (**C**) N-demethylation metabolites of Diuron into DCPMU and DCPU. The methyl groups of the Diuron are circled in red. (**D**) Peak retention times corresponding to Diuron and the three Diuron metabolites (DM1, DM2, and DM3) were observed when incubated with the purified P450 BM3 MT35 with and without NADPH.

In recent years, surface waters have become increasingly polluted by persistent organic contaminants from agricultural and urban runoff, with incompletely treated wastewater being a major source of pesticide, herbicide, and pharmaceutical contaminants (1, 2). Diuron, a phenylamide herbicide commonly used to control weeds in both crop and non-crop areas, is one such contaminant frequently detected in aquatic ecosystems (3). Its widespread use, combined with soil leaching and surface runoff, results in environmental pollution that impacts freshwater systems and groundwater (4, 5). Diuron is an endocrine disruptor with estrogenic and anti-androgenic effects and is classified as a priority hazardous substance by the European Commission (6, 7). Biodegradation of Diuron typically leads to the formation of three primary metabolites: 3,4-dichloroaniline (DCA), 3,4-dichlorophenylurea (DCPU), and 3,4-dichlorophenyl-N-methylurea (DCPMU) (8) (Fig. 1C). However, conventional wastewater treatment processes achieve only partial removal of Diuron, with efficiencies ranging from 70% to 90% (9), leaving trace concentrations (98.2–210 ng/L) that often persist in wastewater treatment plants (WWTPs) (10). The European Union (EU) Directive 89/778/EEC establishes a maximum allowable Diuron concentration of 0.1 µg/L in drinking water (11), highlighting the critical need for innovative and sustainable strategies to mitigate Diuron contamination.

Cytochrome P450 enzymes (hereafter P450), a superfamily of heme-containing proteins, are essential for metabolizing a wide array of both endogenous and xenobiotic compounds, including toxic environmental agents (12). Found across a diverse range of organisms, P450s play a pivotal role in detoxifying xenobiotics by converting lipid-soluble compounds into more water-soluble forms for excretion (13). Among the P450s, the bacterial cytochrome CYP102A1, also known as P450 BM3, is particularly well-suited for biotechnological applications due to its high catalytic efficiency and ability to function as a soluble enzyme without the need for membrane anchoring (14). Isolated from *Bacillus megaterium*, P450 BM3 has a fused heme and reductase domain, allowing it to perform monooxygenase reactions autonomously when supplied with NADPH (15). While P450 BM3 primarily hydroxylates fatty acids, it has also been shown to metabolize a broad range of xenobiotics, including pharmaceuticals (16, 17). Mutants of P450 BM3 have been explored for their potential in metabolizing new prescription drugs, with studies demonstrating their capability to produce metabolites like those generated by human P450 metabolism, as well as unique metabolites (18). Among these mutants, MT35 stands out, displaying efficient metabolism of a wide range of substrates, including pharmaceuticals (18). This broad substrate specificity makes the MT35 mutant of P450 BM3 (hereafter P450 BM3 MT35) particularly attractive for applications in the treatment of emerging contaminant-laden wastewater, offering promising prospects for drug and pesticide remediation.

The *in vivo* metabolism of drugs and pesticides by naturally occurring bacteria or fungi is typically slow and requires extended exposure times to achieve significant degradation, making these methods less effective for wastewater treatment. For instance, *Bacillus muralis*, *Bacillus simplex*, and *Bacillus pseudomycoides* have been shown to metabolize Diuron into the metabolites DCPMU and 3,4-DCA, achieving degradation rates of 51% and 54% of 50 mg/L after a prolonged incubation of 46 days—far exceeding the typical water retention time in municipal wastewater treatment facilities (19). Similarly, the white-rot fungus *Trametes versicolor* can metabolize Diuron into DCPMU and DCPU, but this process also takes weeks, limiting its practicality for large-scale bioremediation of Diuron-contaminated water (20). Thus, methods to enhance the inherent capabilities of microorganisms to metabolize Diuron could greatly enhance the ability of endemic microbial populations in WWTPs.

The use of genetically modified microorganisms has emerged as a promising strategy for addressing environmental pollution. *Chlamydomonas reinhardtii* (hereafter *Chlamydomonas*), a unicellular green alga, has been extensively studied for its genetic flexibility, ease of manipulation, and potential for bioremediation (21). *Chlamydomonas* has been shown to degrade various pollutants, including herbicides, through its metabolic pathways (22, 23). Its chloroplast, a prime target for foreign gene expression, exhibits a high capacity for protein production, making it an ideal platform for enhancing metabolic pathways aimed at environmental detoxification (24–26).

In this study, we investigated the potential of genetically engineered *Bacillus megaterium* and *Chlamydomonas* expressing the P450 BM3 MT35 enzyme to metabolize Diuron. We demonstrated the enhanced degradation efficiency of these modified organisms in degrading Diuron compared to wild-type (WT) strains, highlighting the potential of P450-based biocatalysts for addressing pesticide contamination in wastewater (9). Our findings provide a foundation for future applications of genetically modified microorganisms in the sustainable remediation of emerging environmental contaminants.

## MATERIALS AND METHODS

### Strains and chemicals

*Bacillus megaterium* (BM, ATCC 14581) and *Escherichia coli* BL21 cells were obtained from Invitrogen. The *Chlamydomonas* strains used in this study were CC-400 (WT) and CC-4388 (chloroplast mutant) from the Chlamydomonas Resource Center. Plasmids pET28A+ and pMM1522 were purchased from Millipore Sigma (Novagen) and Boca Scientific, respectively. pASapI was obtained from the Chlamydomonas Resource Center. P450 BM3 MT35 DNA was synthesized and subcloned into pUC57 mini by GenScript Biotech. High-performance liquid chromatography (HPLC)-grade acetonitrile and water were from Caledon Laboratories. Isopropyl ß-D-1-thiogalactopyranoside (IPTG), δ-aminolevulinic acid, thiamine, FeSO_4_, DNaseI, lysozyme, imidazole, sucrose, ammonium sulfate, Diuron, DCPU, and 3,4-DCA were purchased from Millipore Sigma, while DCPMU was obtained from Toronto Research Chemicals. Ni-NTA Agarose was sourced from Qiagen.

### DNA constructs

#### P450 BM3 and P450 BM3 MT35 plasmid construction for *E. coli* expression

Plasmid constructs for P450 BM3 (GenBank: J04832.1) and the mutant P450 BM3 MT35 (R47L, E64G, F81I, F87V, E143G, L188Q, Y198C, E267V, H285Y, G415S, L437S) were subcloned into the pET28A+ vector using BamHI and XhoI restriction sites (Fig. S1). BamHI and XhoI were added before the start and stop codons, with six histidines after the start codon for N-terminal His-tag purification via a Nickel column. *E. coli* BL21 cells were transformed with the above constructs, and positive clones were selected based on antibiotic resistance, plasmid digestion, and sequencing.

#### P450 BM3 MT35 plasmid construction for B*acillus megaterium* expression

The DNA sequence for P450 BM3 MT35 (see amino acid sequence in Fig. S2) was synthesized with a BsrGI site (TGTACA) at the N-terminus before the ATG start codon and a BamHI site after the TAA stop codon. Six CAC codons were added after ATG to create an N-terminal methionine-His6 tag for Nickel column purification, and the sequence was subcloned into pUC57 mini. Following restriction with BsrGI and BamHI, the P450 BM3 MT35 fragment was ligated into pMM1522. Positive clones were confirmed by restriction analysis and sequencing.

#### Plasmid design for *Chlamydomonas* expression

The pASapI vector, described in Economou et al. (27), was used for the introduction of the CYP BM3 MT35 coding sequence into the chloroplast genome. The P450 BM3 amino acid sequence (GenBank WT CYP102 sequence ID: J04832.1) with the mutations described for MT35 by Reinen et al. (18) was codon optimized for the *Chlamydomonas* chloroplast expression using the Chlamy Sequence Optimizer software (https://www.energylab.sites.tau.ac.il/cso) (28) (Fig. S3). The optimized sequence was synthesized using GenScript (Piscataway, USA). The gene was cloned into pASapI using the restriction enzymes SapI and SphI (New England Biolabs) to create pASapIP450BM3 MT35 (Fig. S4).

### Bacterial and algae transformation

#### *Bacillus megaterium* transformation

Protoplasts of *Bacillus megaterium* were prepared by growing cells in an LB medium at 37°C and 250 rpm to an OD^578^ nm of 1. Cells were centrifuged, resuspended in 5 mL freshly prepared SMMP, and treated with SMMP-lysozyme solution at 37°C with gentle shaking until 80%–90% were protoplasted. The protoplasts were collected by centrifugation, washed with SMMP, and stored in 87% sterile glycerol at −80°C. To transform, 500 µL of thawed protoplasts were mixed with 10–20 µL of plasmid DNA (150 ng/mL) and then incubated in PEG-P for 2 min. After centrifugation and washing, cells were resuspended in SMMP, incubated for 90 min (45 min with shaking), and transferred to CR5-top-agar. The mixture was poured onto pre-warmed LB agar plates with tetracycline and incubated at 30°C for 24 h. A single colony was selected for use in this study. Detailed protocol is provided in supplementary methods.

#### Algae chloroplast transformation and confirmation of the mutation at the DNA and RNA levels

The vector pASapI was employed to introduce the gene of interest and restore photosynthetic growth. The Cp transformation strategy is presented in Fig. S4. Cp transformation was carried out according to Economou et al. (27) (more details in Fig. S4). The transgenic lines were subjected to PCR analysis to confirm correct integration of the P450 BM3 MT35 into the Cp genome. Primers were specifically designed to amplify the full-length cDNA, employing overlapping primer pairs (F1 × R1: 1,685 bp; F2 × R2: 1,456 bp) from transformed lines. The gene expression was confirmed at the RNA level by performing semi-quantitative PCR. The RNA extraction was performed as described previously (29). The total RNA of each mutant was reverse transcribed into cDNA using an oligo-dT primer (ThermoFisher Scientific). All the primer sequences are listed in Table S1. Two representative lines were chosen and termed CrM1 and CrM2, together with a pASapI transformant as a negative control.

### Culture conditions

#### Culture, protein preparation, and purification of BM3 MT35 in *E. coli*

BL21 cells containing pET28A(+)P450 BM3 (WT or MT35) were grown overnight at 37°C in 5 mL LB with kanamycin (30 µg/mL). The next day, 500 mL of Terrific Broth (TB) was inoculated with 1 mL of the culture and grown to OD_600_ 0.8. The temperature was lowered to 28°C, agitation to 180 rpm, and IPTG (1 mM), δ-aminolevulinic acid (1 mM), thiamine (1 mM), and FeSO_4_ (1 mM) were added. After 18 h, cells were harvested by centrifugation (8,000 × *g*, 30 min). The pellets were resuspended in phosphate buffer with protease inhibitors, DNase I, and lysozyme, then sonicated. The lysate was centrifuged at 10,000 × *g* and again at 100,000 × *g*. The 100,000 × *g* supernatant was mixed with 10 mM imidazole and purified on a Ni-NTA column. Proteins were washed with 20 mM imidazole and eluted with 300 mM imidazole. After overnight dialysis in phosphate buffer (pH 7.4), proteins were assayed. Detailed protocol is provided in the supplementary methods.

#### Algae culture conditions

Before experiments, strains (CC-400 [WT] and CC-4388) were isolated to monocultures, and the CC-4388 mutation was confirmed by genotyping PCR (Table S1) as per Wannathong et al. (30). CC-400 cells were maintained at ~100 µmol photons m−2 s−1 and 23°C on tris acetate phosphate (TAP) medium (31), while CC-4388 cells were grown under low light (~20 µmol photons m−2 s−1) on TAP supplemented with 100 mg/mL spectinomycin. Transformations were performed with CC-4388 cells, which were then plated on “high salt minimum” media (www.chlamy.org/media.html) and maintained under medium light (~100 µmol photons m−2 s−1) at 23°C. All media plates contained 1.5% Bacto agar. Diuron (Millipore Sigma Canada) was dissolved in acetone at a 40 mM stock concentration, and liarozole (Cayman Chemical) was dissolved in DMSO at 2.5 mg/L. For plate cultures, cells were resuspended in TAP at 1 × 10^5^ cells/mL in 24-well plates. Diuron was diluted from a 40 mM stock to 5, 15, 25, 30, and 50 µM concentrations. In liquid cultures, cells were inoculated at 1 × 10^5^ cells/mL in 100 mL. Samples (500 µL) were collected on days 3, 5, and 7 for cell counts using a hemocytometer. Liarozole was used at 2 µg/mL (6.5 µM) for incubations.

### *E. coli in vitro* metabolism

Purified P450s were incubated with different substrates in borosilicate glass tubes (10 mm × 75 mm) at 22°C, with constant shaking at 220 rpm on an orbital shaker. The reaction mixture (200 µL total) included purified P450 (various concentrations), 20 µL of NADPH (5 mM), 20 µL of a regenerating system (3 mM glucose-6-phosphate and 4 units/mL glucose-6-phosphate dehydrogenase, the substrate (various concentrations), and 0.1 M phosphate buffer. Reactions were stopped by adding 500 µL of cold acetonitrile/methanol (2:1), vortexed, and centrifuged at 8,000 × *g* for 10 min. The supernatant was then used for HPLC analysis.

### HPLC conditions used in *Bacillus megaterium* experiments

Samples were analyzed by HPLC using Agilent technologies 1200 series instruments. A C18 column (Zorbax 100 × 4.6 mm 3.5-Micron, Agilent) and a mobile phase comprised of a mix of acidified (0.1% formic acid) acetonitrile and water were used. A gradient from 1% to 99% acetonitrile in 20 or 30 min with a flow rate of 0.5 mL/min was used to separate Diuron and their metabolites, respectively. The column was maintained at 25°C. Between injections, the column was washed with 99% acetonitrile for 5 min and then returned to 1% acetonitrile over an isocratic 5 min gradient. The absorbance of each compound (Diuron, DCPU, DCPMU, and 3,4-DCA at 243 nm) was used for quantification.

### Synthetic and activated sludge wastewater composition

Synthetic wastewater was prepared following the recipe from El Moussaoui et al. (32), consisting of a Maggi cube, 0.486 g sucrose, 0.41 g ammonium chloride, and 0.061 mL phosphoric acid, dissolved in 5.5 L double distilled water, with the pH adjusted to 7.2 to mimic the characteristics of second digest effluent in wastewater treatment plants and autoclaved. Actual wastewater samples were collected from the run-off facility from the Ravensview (Kingston, ON, Canada) and Alexandria (North Glengarry, ON, Canada) water treatment facilities in 2022, post-secondary digestion. These samples were autoclaved for 24 h and subsequently inoculated with cultures without further treatment.

### *In vivo* metabolism

#### Liquid cultivation of *Bacillus megaterium* and *in vivo* metabolism

Liquid cultivation for *in vivo* drug metabolism was conducted at 22°C with constant shaking (180 rpm) in TB or wastewater. For TB cultures, 1 mL of overnight LB culture was added to 20 mL TB containing tetracycline (15 µg/mL) for transformed *Bacillus megaterium* (tBM). Cells were grown to an OD^578^ of 0.3, induced with 0.5% xylose, and 10 µM substrate was added after 3 h. Samples (200 µL) were taken over a 96 h period, treated with acetonitrile/methanol (2:1), and analyzed by HPLC. For wastewater studies, a 500 mL TB culture was grown to OD^578^ 0.3–0.4, induced with xylose, then centrifuged and resuspended in TB. A total of 3 mL of the resuspended cells were added to 20 mL of synthetic (SWW) or municipal (MWW) wastewater with 10 µM substrate, maintaining tetracycline in all tBM cultures.

#### Diuron removal efficiency in algae

To measure Diuron removal efficiency, 2 mL of each culture was collected at day 0, day 3, day 5, and day 7. All the samples were spun at 4,200 *× g* for 5 min, and the supernatant was collected and filtered through a 0.45 µM nitrocellulose filter (Mandel Scientific) before the Diuron measurements. Samples were analyzed using an ISQ EM Single Quadrupole Mass Spectrometer in positive ion mode. The ion transfer tube temperature was set to 300°C. An ion spray voltage of 3.0 kV was applied to the ESI emitter. A C18 column (Hypersil ODS 50 × 4.6 mm 3-Micron, Thermo Scientific) and a mobile phase comprised of 100% acetonitrile were used. An isocratic gradient over 5 min with a flow rate of 0.4 mL/min was used to separate Diuron. The column was maintained at 25°C. The Diuron area in each of the mass spectrometer-generated plots was used in the analysis and % Diuron removal efficiency in each sample.

### Statistical analysis

All assays were performed in at least three independent experiments. Data were expressed as mean  ±  SD. The GraphPad Prism 7 software (GraphPad Software, La Jolla, CA, USA) was used for statistical analysis. Two-tailed Student’s *t*-tests were performed to evaluate the differences among groups. Differences among means were considered significant at *P* ≤ 0.05.

## RESULTS

### P450 BM3 MT35 expression in *E. coli* and *in vitro* metabolism

The presence of the active P450 BM3 MT35 was confirmed following the method by Omura and Sato (33), based on the reaction between the ferrous form of the heme protein and carbon monoxide, resulting in a specific absorption peak at 450 nm (Fig. 1A). The purified enzyme was further characterized by SDS-PAGE, showing a molecular weight of 118 kDa, significant to P450 BM3 (Fig. 1B). Similar results were observed for WT P450 BM3, which was used as a reference (data not shown) confirming that P450 BM3 MT35 was effectively expressed and capable of complexing with heme.

*In vitro* assays with P450 BM3 MT35 and NADPH revealed the formation of two major metabolites (DM1: RT 14 min and DM2: RT 13 min) and one minor metabolite (DM3: RT 12.2 min) (Fig. 1D). No metabolites were detected when WT P450 BM3 was used, even at high substrate concentrations up to 1 mM (data not shown). Additionally, control incubations without NADPH did not yield any metabolites, indicating that the observed metabolism was P450-dependent. After 5 min of incubation, the primary metabolite was the RT 13 min peak (DM2). However, after 60 min, the RT 14 min peak (DM1) became the dominant product.

To identify whether these are the demethylated metabolites of Diuron (Fig. 1C), commercial reference compounds DCPMU, DCPU, and DCA were used, and their retention times were compared with the P450 BM3 MT35 products in HPLC. The RT 14 min metabolite (DM1) was identified as the N-demethylated DCPMU, while the RT 13 min metabolite (DM2) matched the di-N-demethylated DCPU. No 3,4-DCA was detected, even at substrate concentrations as high as 1 mM, and the identity of the minor product (DM3) remains unknown and requires further investigation for identification.

### *In vivo* metabolism of Diuron by the transgenic *Bacillus megaterium* cells

**Fig 2:**
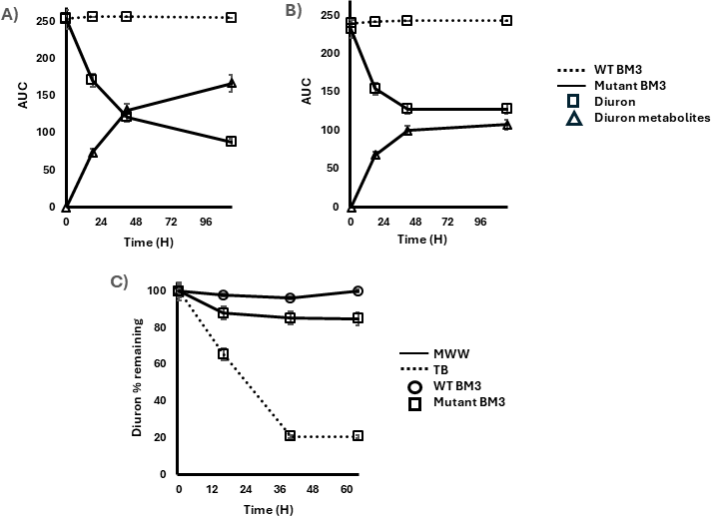
Metabolism of Diuron in TB medium (**A**), SWW (**B**), and MWW (**C**). In TB medium (**A**), Diuron is rapidly metabolized by the mutant P450 BM3 MT35 lines, with 23% of the initial 10 mM Diuron metabolized after 24 h of incubation. By day 5, 35% of the Diuron remained intact, and metabolism had nearly ceased. The control cultures with WT *Bacillus megaterium* did not produce any metabolites. Similar patterns were observed in SWW (**B**), where Diuron was metabolized up to 45% before reaching a plateau after 5 days. As in the TB medium, the decrease in Diuron in SWW correlated with an increase in its metabolites. In MWW samples, Diuron metabolism occurred, reaching 15% after 2.5 days of incubation (**C**). Data are expressed as the mean ± standard deviation.

*In vivo* metabolism of Diuron was analyzed by comparing WT *Bacillus megaterium* with one of their transformed counterparts. As shown in Fig. 2A, in TB medium, Diuron was rapidly metabolized by the transgenic strain, with 23% of 10 µM Diuron degraded after 24 h. By day 5, 35% of Diuron remained, indicating that metabolism had nearly ceased. This plateau is likely due to the transient expression of P450 BM3 MT35, which appears to persist for 48–72 h. Control cultures of WT *Bacillus megaterium* did not produce any metabolites. The primary metabolite identified was DCPMU, while DCPU and the unknown metabolite, which likely is the result of hydroxylation, accounted for less than 15% of total metabolism (data not shown). A similar trend was observed in SWW (Fig. 2B), where Diuron was metabolized up to 45%, with degradation plateauing after 5 days. As in the TB medium, the decline in Diuron was associated with an increase in Diuron metabolites, primarily DCPMU, and no detectable metabolism occurred in WT Bacillus megaterium cultures. In MWW samples (Fig. 2C), Diuron metabolism reached 15% after 2.5 days with DCPMU as the primary metabolite. Compared to the optimal conditions provided in the TB medium, where 80% of Diuron was metabolized within the same timeframe, metabolism in MWW was also significant but reduced compared to TB controls. This reduced metabolism is likely attributed to the static growth conditions, which can limit nutrient availability and oxygen diffusion, leading the bacteria to prioritize essential metabolic processes for survival rather than dedicating energy toward Diuron degradation.

### Expression analysis of the recombinant P450 BM3 MT35 in *Chlamydomonas*

**Fig 3:**
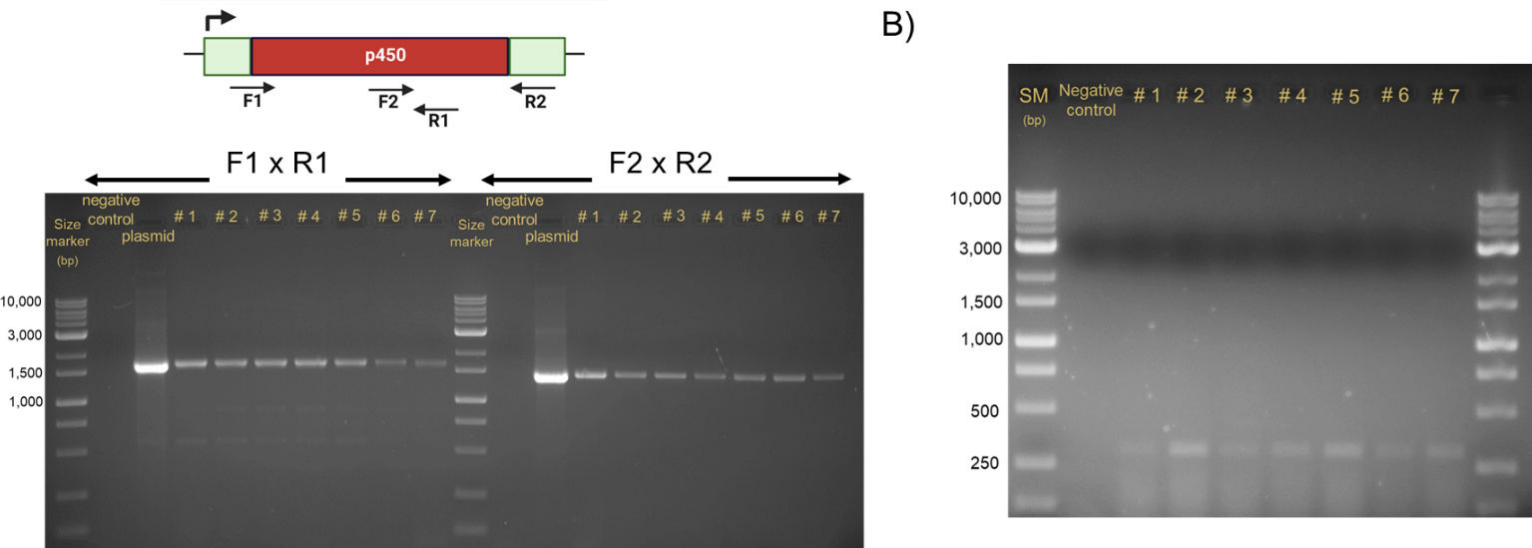
Confirmation of the P450 BM3 MT35 mutants in *Chlamydomonas reinhardtii*. (**A**) Genomic DNA extracted from seven of the transformed algae cultures was subjected to PCR analysis using overlapping primer pairs F1 × R1 and F2 × R2 to assess integration of the P450 BM3 MT35 construct. Untransformed cells (negative control) and the pASapIP450BM3 MT35 plasmid used in transformation (positive control) were also analyzed. (**B**) Expression of P450 BM3 MT35 in the chloroplast of *Chlamydomonas*. Total RNA samples from TN72 and P450 BM3 MT35 transformant strains underwent semi-quantitative PCR analysis. Transformants exhibited amplification at 26 cycles, indicating the expression of P450 BM3 MT35 in these lines.

The transformation of *Chlamydomonas* chloroplast with pASapIP450BM3 MT35 yielded seven positive colonies. PCR analysis of transgenic lines (#1 to #7) revealed clear bands for both primer pairs, as depicted in Fig. 3A. Conversely, the negative control, consisting of the empty pASapI vector, did not produce any bands. The pASapIP450BM3 MT35 vector utilized in the transformation served as the positive control.

The expression of P450 BM3 in the *Chlamydomonas* chloroplast was investigated through semi-quantitative PCR analysis using cDNA generated from the total RNA samples. These samples were obtained from two groups: the first comprised *Chlamydomonas* transformants containing only the pASapI vector without P450 BM3 MT35 (negative control in Fig. 3B), while the second included seven transformant strains harboring P450 BM3 MT35 (#1 to #7 in Fig. 3B).

Upon PCR amplification, transformants displayed detectable amplification signals at 26 cycles, indicative of gene expression (Fig. 3B). In contrast, samples from the control group lacking P450 BM3 MT35 did not exhibit amplification signals, affirming the specificity of the detected expression signals in the P450 BM3 MT35 transformants. Further experiments were carried out using two of the P450 BM3 MT35 transgenic lines (CrM1 and CrM2).

### CrM1 and CrM2 transgenic lines showed improved resistance to Diuron

**Fig 4:**
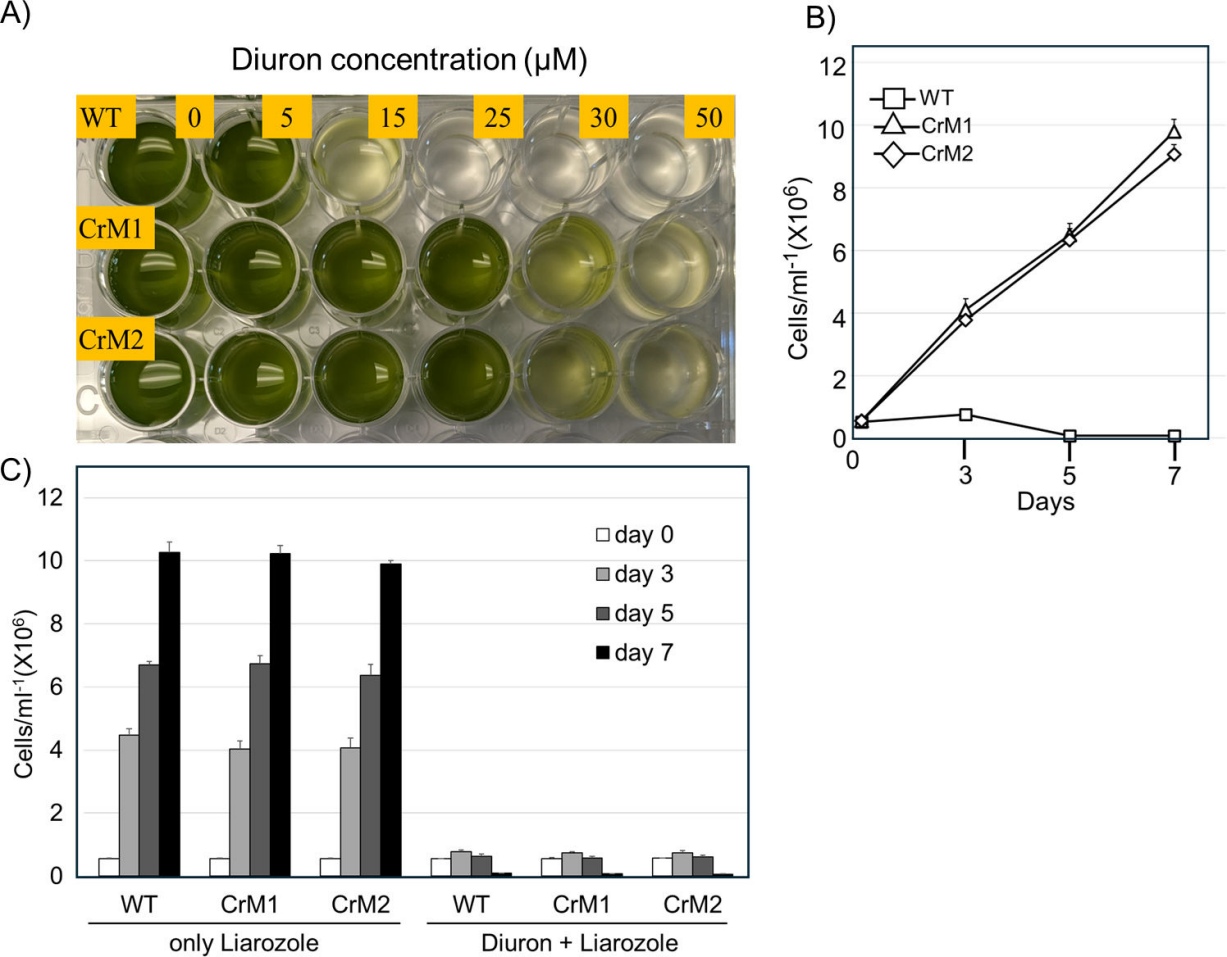
Resistance of the P450 BM3 MT35 lines to Diuron. (**A**) WT and P450 BM3 MT35 lines (CrM1 and CrM2) were exposed to different Diuron concentrations. EC_90_ of the WT cells (measured using hemocytometer-based cell counts) reached 25 µM, and the EC_50_ of the P450 BM3 MT35 lines were reached at 50 µm Diuron. (**B**) Growth curves (mean ± standard error) of the WT and the P450 BM3 lines at 25 µM Diuron concentration. The growth was significantly different between WT and P450 BM3 lines (*P*-values < 0.0001), but no significant differences were observed between CrM1 and CrM2. (**C**) Growth curves (mean ± standard error) of WT and P450 BM3 lines at 25 µM Diuron and 20 µg/mL Liarozole. Significant differences in growth were observed only between Liarozole and Diuron + Liarozole treatments across all tested lines (*P*-values < 0.0001). However, no significant differences were found among WT, CrM1, and CrM2 lines under Liarozole or Diuron + Liarozole conditions.

To evaluate potential variations in Diuron survival among transgenic lines, we assessed the growth of two transgenic lines, CrM1 and CrM2, across a range of Diuron concentrations spanning from 5 µM to 50 µM. Our observations revealed enhanced survival or resistance of the P450 BM3 lines to Diuron compared to WT cells. Notably, the EC90 value for WT cells was reached at 25 µM Diuron, while the EC50 for the P450 BM3 MT35 lines was reached at 50 µM Diuron (Fig. 4A). Further examination of growth curves at a Diuron concentration of 25 µM demonstrated that while WT cells exhibited stagnant growth, transgenic lines displayed exponential growth. This discrepancy suggests that the expression of P450 BM3 MT35 contributes to improved survival in high Diuron concentrations (Fig. 4B).

**Fig 5:**
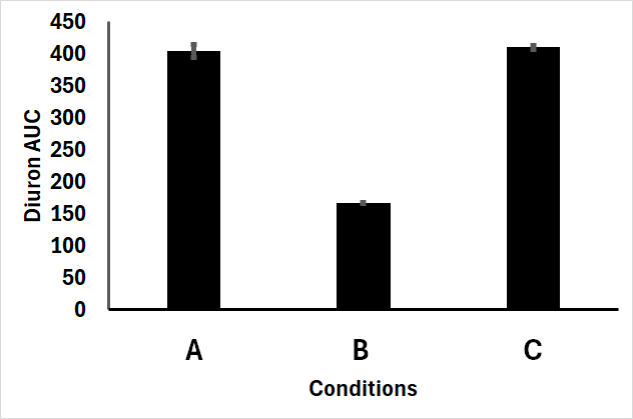
*In vitro* BM3 MT35 inhibition by Liarozole. A 25 µM Diuron was incubated with BM3 MT35 for 1 h at room temperature in the absence of NADPH and Liarozole (A), in the presence of NADPH and absence of Liarozole (B), and finally in the presence of NADPH and 2 µg/mL Liarozole (C). Data show remaining Diuron present in the incubation (AUC, area under the curve) and are expressed as the mean ± standard deviation.

To confirm the role of P450 BM3 MT35 expression in this enhanced survival, we conducted additional experiments using a broad-range P450 inhibitor, liarozole. Since wild-type P450 BM3 has very limited inhibition through imidazoles, *in vitro* inhibition studies of Diuron metabolism by BM3 MT35 with liarozole were conducted. In contrast to wild-type BM3, BM3 MT35 activity is highly sensitive to liarozole (Fig. 5). When exposed solely to liarozole, cell growth remained unaffected. However, in the presence of both liarozole and 25 µM Diuron, transgenic lines exhibited defective growth comparable to the WT, indicating that active P450 BM3 MT35 expression in the transgenic lines enables survival in high Diuron concentrations (Fig. 4C).

### CrM1 and CrM2 transgenic lines showed enhanced degradation of Diuron

**Fig 6:**
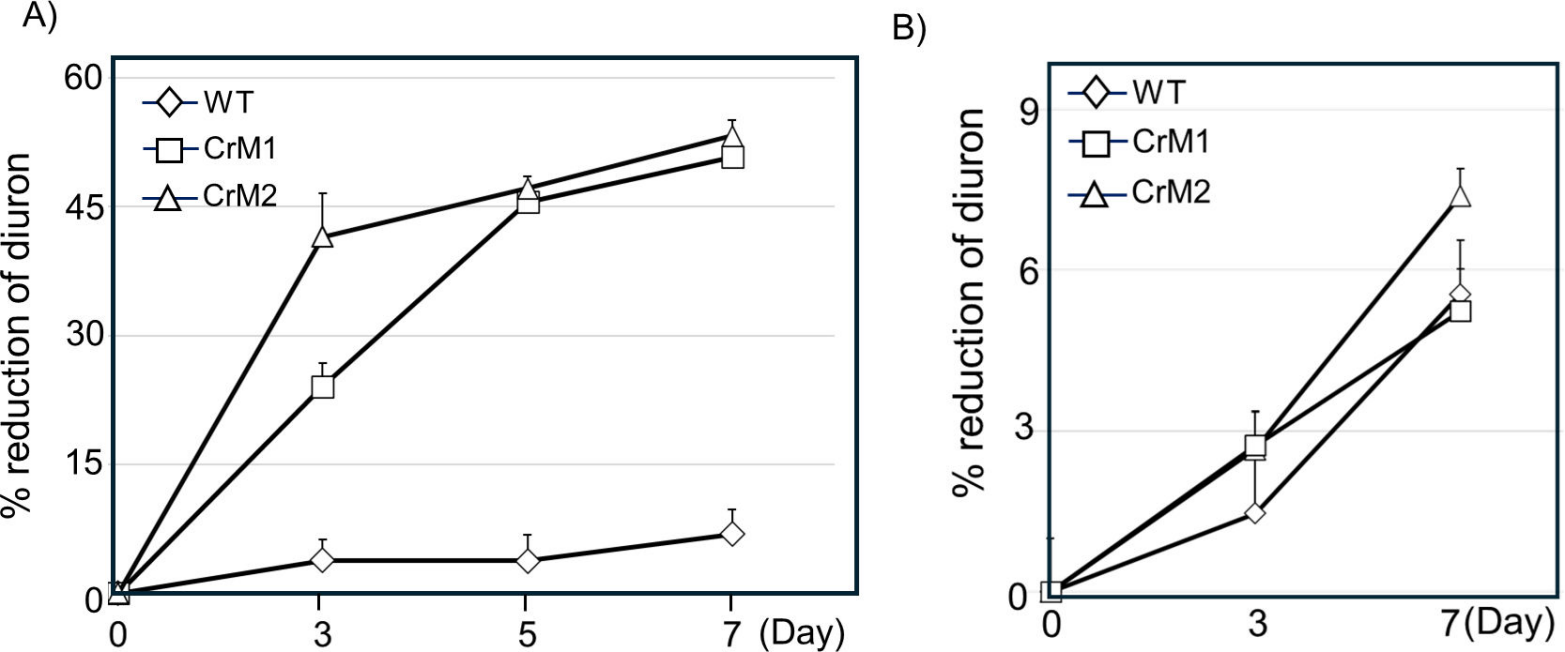
Diuron degradation in P450 BM3 MT35 lines. (**A**) Reduction (mean ± standard error) of Diuron in WT and P450 BM3 MT35 lines at 10 µM Diuron concentration. Significant differences in % reduction of Diuron were observed only between WT and P450 BM3 MT35 lines at day 7 (*P*-values < 0.0001). However, no significant differences in % reduction of Diuron were observed among the CrM1 and CrM2 lines at day 7. (**B**) Reduction (mean ± standard error) of Diuron in the presence of the P450 inhibitor, Liarozole. No significant differences were found in % reduction of Diuron among the WT, CrM1, and CrM2 lines at day 7.

To assess the Diuron degradation efficiency in transgenic lines expressing P450 BM3 MT35, we exposed both WT cells and transgenic CrM1 and CrM2 lines to 10 µM Diuron and monitored Diuron levels on days 3, 5, and 7 (Fig. 6A). Strikingly, by day 7, CrM1 and CrM2 exhibited 50.8% and 53.3% Diuron degradation, respectively, whereas WT cells only showed 6.9% degradation. Moreover, in the presence of liarozole, the Diuron degradation capabilities of both transgenic lines declined, with degradation rates comparable to the WT (Fig. 6B), suggesting that the functional P450 BM3 MT35 in the transgenic lines enhances Diuron degradation.

The substantial increase in both the amount and rate of Diuron degradation observed in transgenic lines expressing P450 BM3 MT35 highlights the potential of genetic engineering for enhancing bioremediation capabilities in microalgae. The enzymatic activity of P450 BM3 MT35 likely facilitates the breakdown of Diuron, leading to improved degradation rates compared to WT cells. This finding aligns with previous studies demonstrating the detoxification capabilities of P450 enzymes in various organisms (34), underscoring the effectiveness of this approach for pollutant remediation. Furthermore, the decrease in Diuron degradation upon inhibition of P450 activity with liarozole reaffirms the pivotal role of P450 BM3 MT35 in enhancing Diuron degradation in transgenic lines. This dependency on P450 activity underscores the importance of maintaining functional enzyme expression for effective pollutant degradation.

## DISCUSSION

Our study demonstrated the successful expression of a P450 BM3 MT35 mutant in both *Bacillus megaterium* and *Chlamydomonas reinhardtii*, significantly enhancing their capacity to metabolize Diuron. *In vitro* assays confirmed the enzymatic activity of the engineered P450 BM3 MT35, leading to the formation of key Diuron metabolites, primarily DCPMU and DCPU (Fig. 1D), while avoiding the formation of the more toxic degradation product, 3,4-DCA (35). *In vivo*, the transgenic *Bacillus megaterium* strain demonstrated efficient Diuron metabolism under optimized conditions in TB medium, achieving 65% degradation after 5 days (Fig. 2A). Although lower degradation rates were observed in SWW and MWW, the engineered bacteria still outperformed the WT strain, underscoring the effectiveness of genetic engineering in enhancing pollutant metabolism in complex environmental matrices (Table 1). The reduced Diuron degradation in MWW could be attributed to nutrient limitations and suboptimal growth conditions, which have been shown to affect bacterial metabolism in previous studies (36, 37). Nonetheless, the ability of the engineered *Bacillus megaterium* to metabolize Diuron in real-world water matrices highlights its potential for biotechnological applications in wastewater treatment.

**TABLE 1:** Comparison of degradation methods for Diuron removal

Method	Degradation rates	Efficiency	Practical scalability	Reference
Microbial consortia	Variable	32.2% (in 54 days)–99.8% (in 100 days)	Long treatment times	Ellegaard-Jensen et al. (38); Villaverde et al. (39)
Immobilized enzymes	High	65% (in 16 h)	High cost of enzyme production and immobilization	Ulu et al. (40)
Photodegradation	High	100% (in 20 min), 100% (in 6 h)	Highly expensive due to its energy-intensive nature and reliance on photocatalysts like TiO₂	Katsumata et al. (41); 42
Using genetically modified bacteria and algae	High	65% (in 5 days) for bacteria, 52% (in 7 days) for algae	Cost-effective with potential for large-scale applications	This study

Another key advancement of this study is the expression of P450 BM3 MT35 in the chloroplasts of *Chlamydomonas reinhardtii*. The transgenic CrM1 and CrM2 lines exhibited significantly improved Diuron degradation compared to wild-type strains, degrading over 50% of the herbicide after 7 days (Fig. 6A). This enhanced degradation, coupled with increased resistance (Fig. 4 and 6) to Diuron at concentrations up to 50 µM, suggests that the chloroplast-localized P450 BM3 MT35 facilitates efficient pollutant breakdown. Additionally, the ability of photosynthetic organisms like *Chlamydomonas* to provide the necessary reducing power for P450 enzyme activity offers a sustainable, energy-efficient platform for bioremediation applications. This approach could be especially valuable for large-scale wastewater treatment systems, where continuous degradation of organic pollutants is needed.

Diuron metabolism by BM3 MT35 produces metabolites that have been shown in different systems to have different toxicity than parent compounds. For example, DCPU showed more cytotoxicity than Diuron in Caco-2 cells; however, DCPU was less toxic than Diuron using BeWo cells (43). These data illustrate the complexity of defining the toxicity of Diuron metabolites, which is dependent on the environment and the type of organism targeted. Here, we demonstrated that P450 BM3 MT35 produces three metabolites and that the same enzyme transformed in *Chlamydomonas reinhardtii* confers resistance to Diuron (Fig. 4), suggesting that these metabolites are less toxic in algae or are further metabolized by phase II enzymes.

The dual approach of utilizing both bacterial and algal systems for Diuron degradation underscores the complementary strengths of these organisms for bioremediation. While *Bacillus megaterium* is not commonly present in WWTPs (44), its capacity to use diverse carbon sources and withstand a broad temperature range (3°C to 45°C) (45) makes it an attractive candidate for wastewater treatment applications. However, given the environmental risks associated with bacterial systems, such as uncontrolled proliferation or the spread of antibiotic resistance genes, using them in controlled bioreactor setups is a safer and more effective strategy. In a complementary approach, *Chlamydomonas reinhardtii* offers an alternative, sustainable, and scalable solution for Diuron degradation, particularly in outdoor or large-scale water treatment applications. Numerous microalgae species, such as *Chlamydomonas reinhardtii*, *Anabaena cylindrica*, *Chlorella vulgaris*, *Scenedesmus obliquus*, and *Spirulina platensis*, have demonstrated the potential for cultivation in various wastewaters (46, 47). Moreover, *Chlamydomonas* sp. have proven effective in removing a wide range of wastewater contaminants, including pharmaceuticals such as Diclofenac (23), Enrofloxacin (48), and β-estradiol (49), due to their adaptability to harsh growth conditions (50, 51).

Scaling up the industrial application of genetically modified algae poses several challenges, including maintaining stable enzyme expression, optimizing reactor design, and ensuring seamless integration into existing WWTP infrastructure (9). Engineered microalgae could be incorporated into systems such as high-rate algal ponds, constructed wetlands, or algal biofilm reactors (52–55). When paired with conventional technologies like activated sludge or biofilters, these integrated approaches can achieve synergistic effects, enhancing the removal of pharmaceutical contaminants while promoting efficient nutrient cycling and microbial oxygenation within WWTPs. Furthermore, addressing critical aspects such as economic feasibility, enzyme stability, and biosafety concerns in open systems is essential for scaling up genetically modified algae applications (9).

Algal immobilization offers a promising solution, providing physical protection for engineered algae against environmental stressors and extending their retention time, thereby improving contaminant degradation efficiency (56). To mitigate biosafety risks, containment strategies similar to those proposed for *B. megaterium*, such as bioreactor-based systems or enclosed cultivation units, could be adapted for algae-based systems. These strategies would help limit environmental exposure and address potential ecological risks associated with genetically modified organisms.

To advance this research, we propose pilot-scale experiments in controlled WWTP environments to evaluate the scalability and practical applicability of P450 BM3 MT35-engineered bacterial and microalgae. Such studies would allow testing under diverse operational conditions, offering critical insights into enzyme efficiency, system durability, and Diuron removal rates. Additionally, exploring regulatory frameworks for the environmental release and safe use of genetically modified algae and bacteria will be vital for compliance and public acceptance. Comprehensive techno-economic assessments are also necessary to compare the cost-effectiveness of these systems with traditional tertiary treatment methods, such as UV irradiation and ozonation (57). Addressing these aspects through targeted research and pilot-scale validation will facilitate the development of scalable, feasible, and environmentally safe P450 BM3 MT35-engineered bacteria and microalgae for industrial wastewater treatment applications.

In conclusion, this study demonstrates the potential of engineered microorganisms expressing P450 BM3 MT35 to degrade the toxin Diuron efficiently, offering a promising solution for the bioremediation of water and soil contaminated by this and potentially other emerging agents. Future research should aim to optimize these systems for large-scale application, exploring strategies to enhance enzyme stability and expression. Overall, the integration of genetic engineering with bioremediation holds great promise for developing sustainable, effective wastewater treatment technologies.

## SUPPLEMENTAL MATERIAL

Fig. S1 spectrum.02905-24-s0001.tif Plasmid construct (bacterial).

Fig. S2 spectrum.02905-24-s0002.tif Protein sequence data.

Fig. S3 spectrum.02905-24-s0003.tif Plasmid construct (algae).

Fig. S4 spectrum.02905-24-s0004.tif Algal transformation process.

Supplemental material spectrum.02905-24-s0005.docx Figure legends; Table S1; Supplemental methods.

ASM does not own the copyrights to Supplemental Material that may be linked to, or accessed through, an article. The authors have granted ASM a non-exclusive, world-wide license to publish the Supplemental Material files. Please contact the corresponding author directly for reuse.
