# Supplementary figures and images for: Genetically engineered bacteria and microalgae expressing a mutant of cytochrome P450 BM3 for efficient Diuron degradation in wastewater treatment
Source: Microbiol Spectr. 2025 Apr 16;13(6):e02905-24. doi: 10.1128/spectrum.02905-24 (PMC12131741; doi:10.1128/spectrum.02905-24)

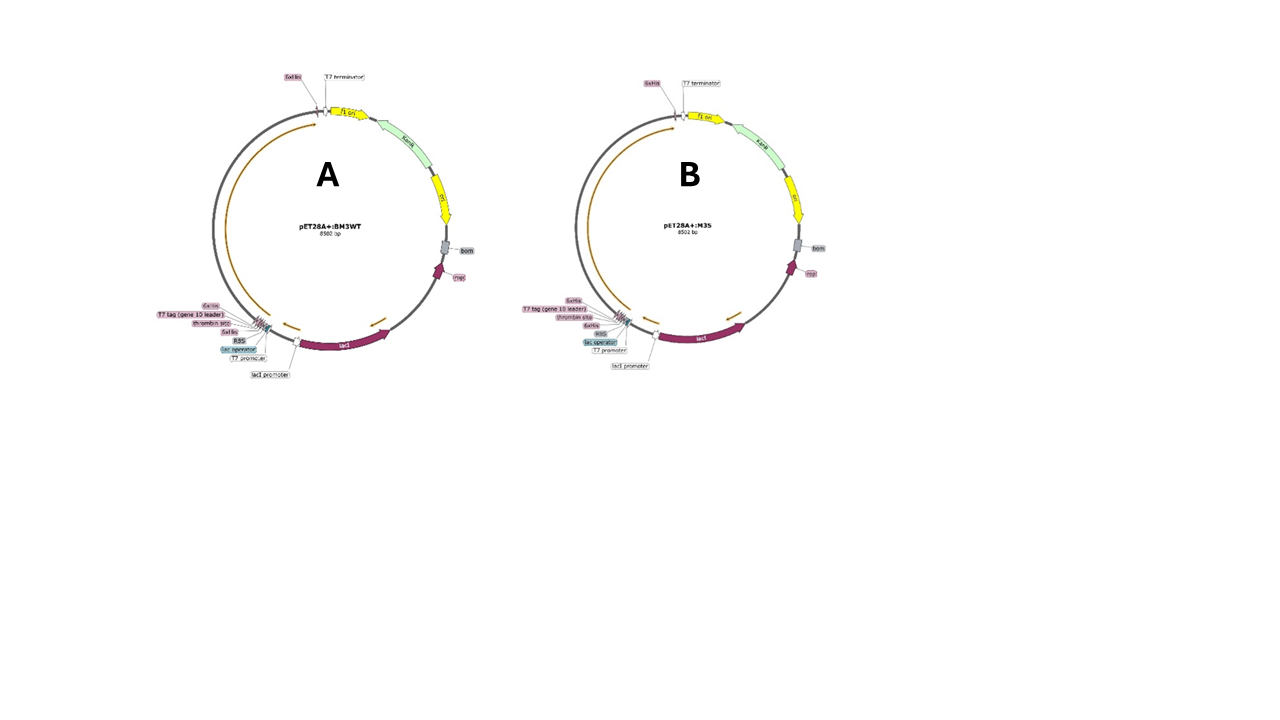

Supplement: Fig. S1 — Plasmid construct (bacterial). [file spectrum.02905-24-s0001.tif]

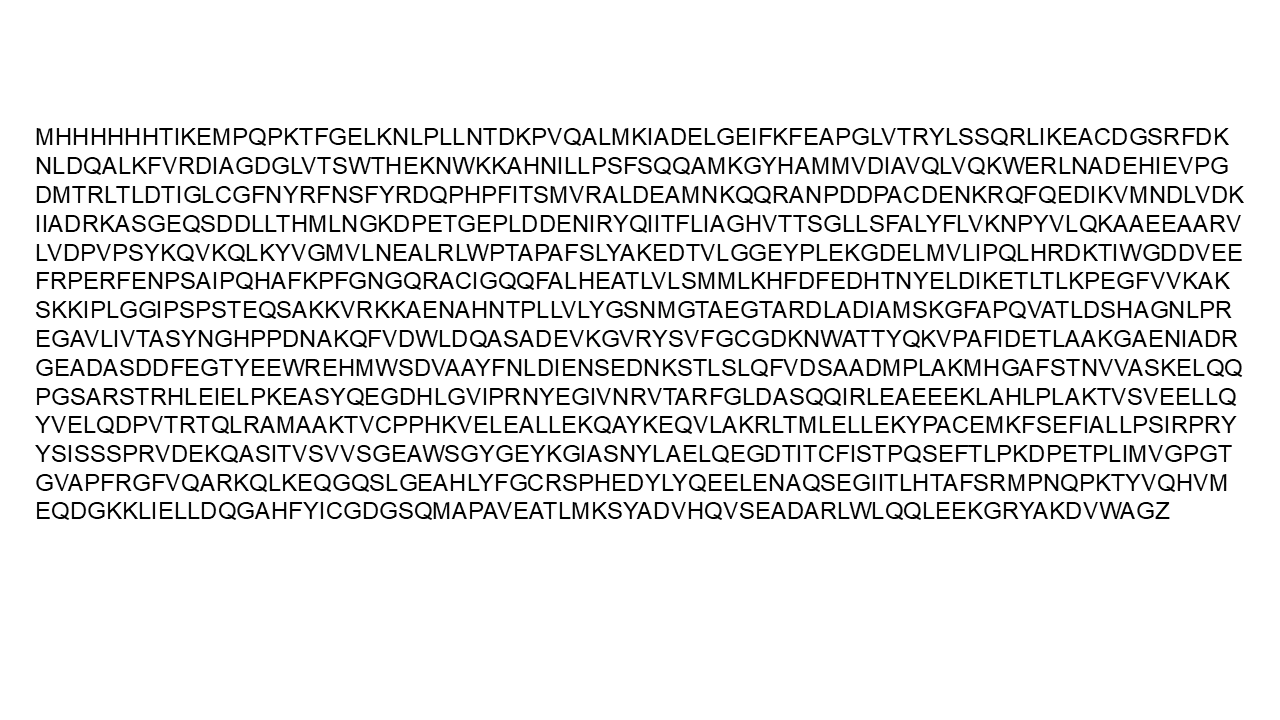

Supplement: Fig. S2 — Protein sequence data. [file spectrum.02905-24-s0002.tif]

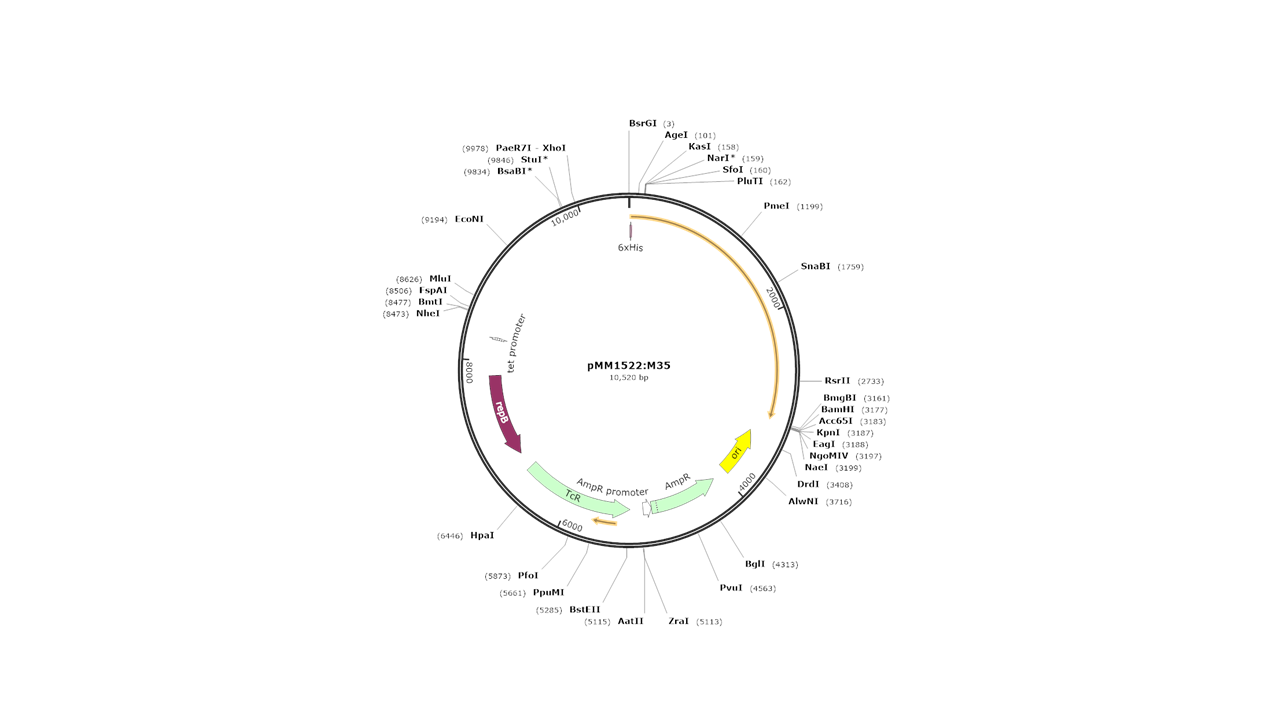

Supplement: Fig. S3 — Plasmid construct (algae). [file spectrum.02905-24-s0003.tif]

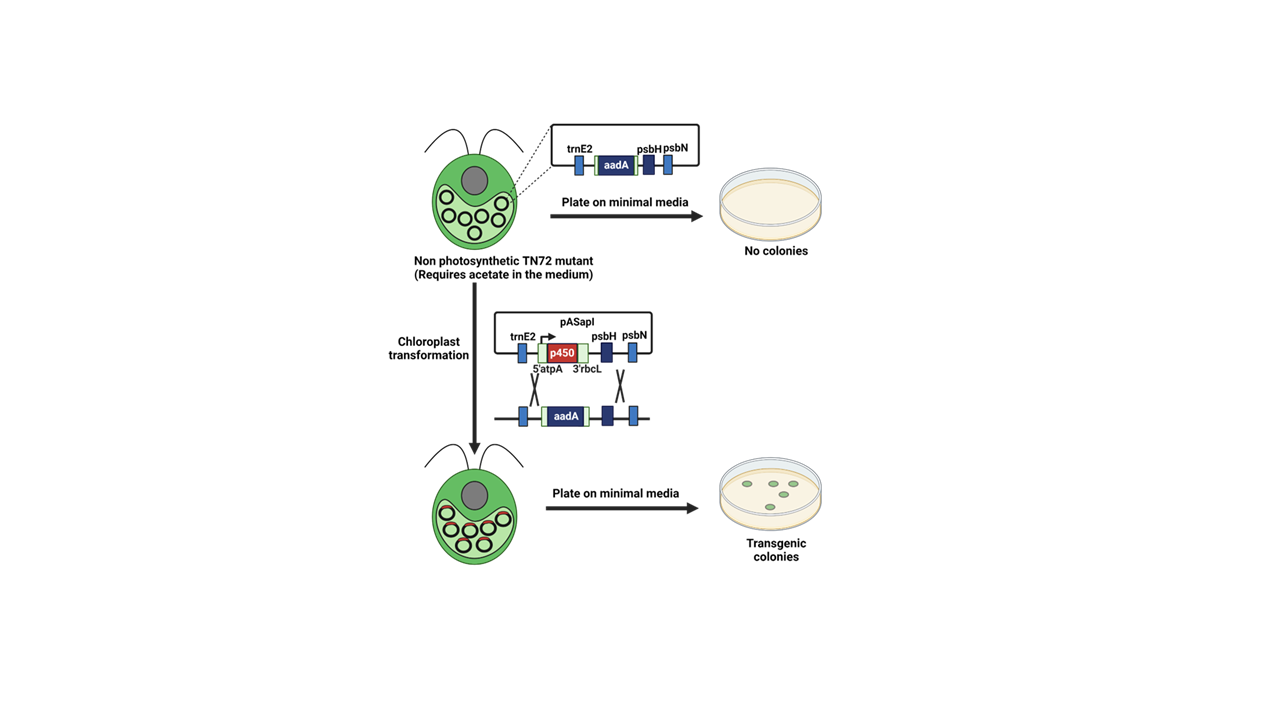

Supplement: Fig. S4 — Algal transformation process. [file spectrum.02905-24-s0004.tif]
