# Supplementary material for: Genetically engineered bacteria and microalgae expressing a mutant of cytochrome P450 BM3 for efficient Diuron degradation in wastewater treatment
Source: Microbiol Spectr. 2025 Apr 16;13(6):e02905-24. doi: 10.1128/spectrum.02905-24 (PMC12131741; doi:10.1128/spectrum.02905-24)
Supplement: Supplemental material — Figure legends; Table S1; Supplemental methods. [file spectrum.02905-24-s0005.docx]

**Supplementary Figures**


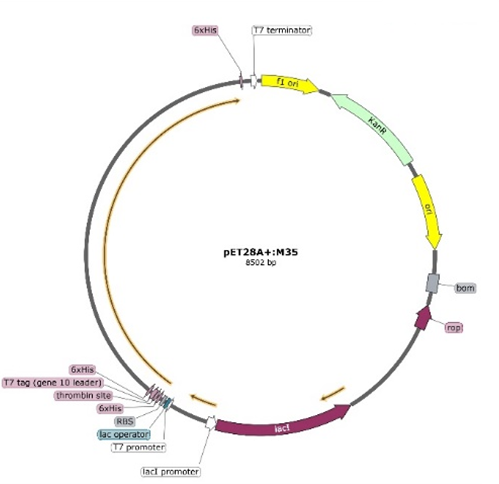

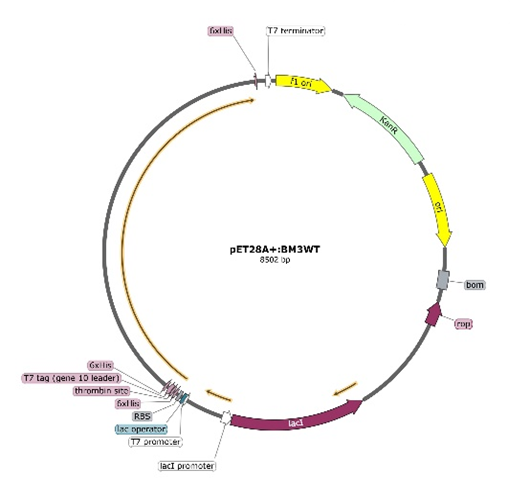
**Figure S1:** **Plasmid constructs for E.coli expression. A-Wild type, B-M35**

**Figure S2:** **M35 Amino acid sequence**

MHHHHHHTIKEMPQPKTFGELKNLPLLNTDKPVQALMKIADELGEIFKFEAPGLVTRYLSSQRLIKEACDGSRFDKNLDQALKFVRDIAGDGLVTSWTHEKNWKKAHNILLPSFSQQAMKGYHAMMVDIAVQLVQKWERLNADEHIEVPGDMTRLTLDTIGLCGFNYRFNSFYRDQPHPFITSMVRALDEAMNKQQRANPDDPACDENKRQFQEDIKVMNDLVDKIIADRKASGEQSDDLLTHMLNGKDPETGEPLDDENIRYQIITFLIAGHVTTSGLLSFALYFLVKNPYVLQKAAEEAARVLVDPVPSYKQVKQLKYVGMVLNEALRLWPTAPAFSLYAKEDTVLGGEYPLEKGDELMVLIPQLHRDKTIWGDDVEEFRPERFENPSAIPQHAFKPFGNGQRACIGQQFALHEATLVLSMMLKHFDFEDHTNYELDIKETLTLKPEGFVVKAKSKKIPLGGIPSPSTEQSAKKVRKKAENAHNTPLLVLYGSNMGTAEGTARDLADIAMSKGFAPQVATLDSHAGNLPREGAVLIVTASYNGHPPDNAKQFVDWLDQASADEVKGVRYSVFGCGDKNWATTYQKVPAFIDETLAAKGAENIADRGEADASDDFEGTYEEWREHMWSDVAAYFNLDIENSEDNKSTLSLQFVDSAADMPLAKMHGAFSTNVVASKELQQPGSARSTRHLEIELPKEASYQEGDHLGVIPRNYEGIVNRVTARFGLDASQQIRLEAEEEKLAHLPLAKTVSVEELLQYVELQDPVTRTQLRAMAAKTVCPPHKVELEALLEKQAYKEQVLAKRLTMLELLEKYPACEMKFSEFIALLPSIRPRYYSISSSPRVDEKQASITVSVVSGEAWSGYGEYKGIASNYLAELQEGDTITCFISTPQSEFTLPKDPETPLIMVGPGTGVAPFRGFVQARKQLKEQGQSLGEAHLYFGCRSPHEDYLYQEELENAQSEGIITLHTAFSRMPNQPKTYVQHVMEQDGKKLIELLDQGAHFYICGDGSQMAPAVEATLMKSYADVHQVSEADARLWLQQLEEKGRYAKDVWAGZ

**Figure S3:** **Plasmid constructs for *Bacillus megaterium* expression**


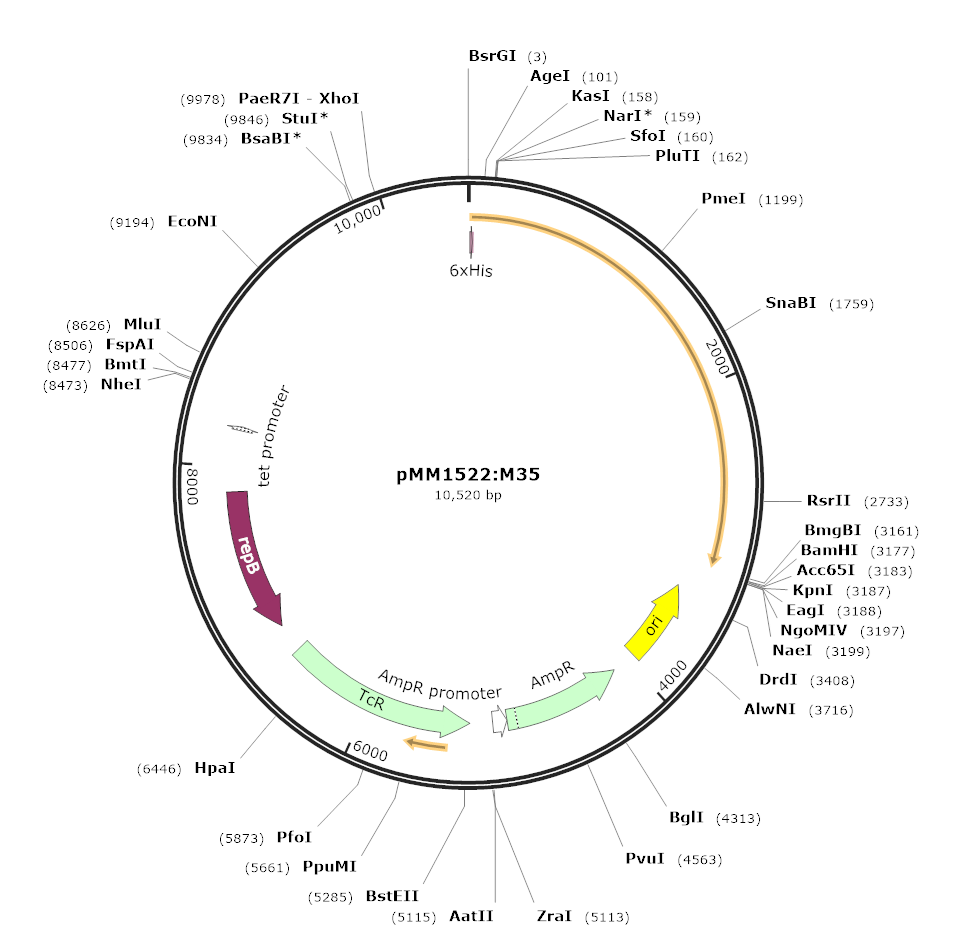


**Figure S4: Cp transformation strategy in *Chlamydomonas reinhardtii***


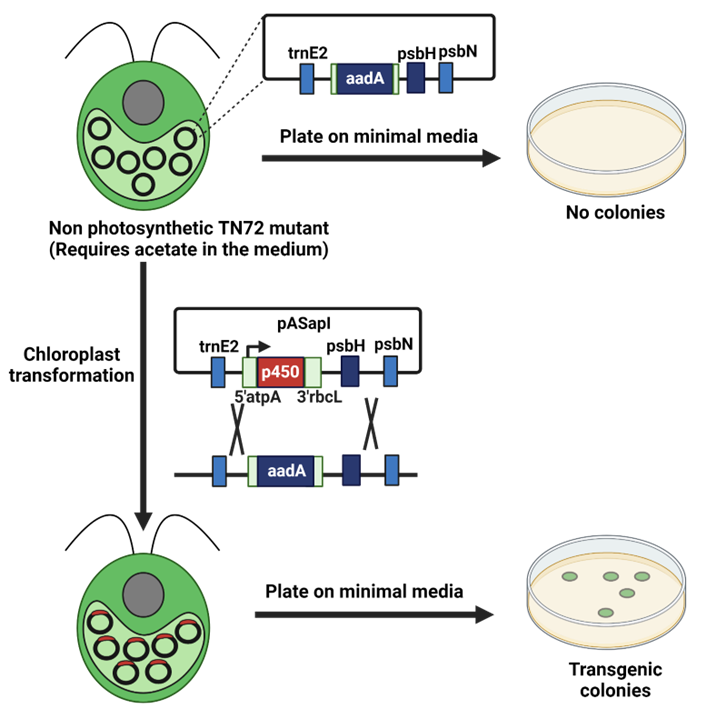


Expression cassette of pASapI includes the promoter, 5` UTR, and start codon of atpA, a multiple cloning site, and the stop codon and 3`UTR of rbcL. Through homologous recombination, the aadA cassette is replaced with both a functional copy of psbH and the gene of interest, in this case, the coding sequence for P450 BM3-1. The codon-optimized coding sequence for P450 BM3-1 intended for expression in the *Chlamydomonas* Cp was synthesized de novo and subsequently cloned into the pASapI vector. Cp transformation was carried out through rapid agitation of a mixture of DNA and cells with glass beads, followed by selection for photoautotrophic growth. After approximately four weeks, colonies of transformed cells were obtained and subjected to subsequent analysis. Control transformations, conducted without the inclusion of DNA, failed to yield any colonies. Importantly, the gene of interest remains the sole segment of foreign DNA in the transformed genome.

**Table S1**

| Primer name | Sequence |
| --- | --- |
| atpA.F | CAAGTGATCTTACCACTCAC |
| rbcL.R | CAAACTTCACATGCAGCAGC |
| P450 BM3-1 F1 | TTTGAAGCTCCAGGTCGTGT |
| P450 BM3-1 R1 | GCTTCACCACGATCAGCAAT |
| P450 BM3-1 F2 | TACCACGTGAAGGTGCTGTTT |
| P450 BM3-1 R2 | CCACGAAATGGAGCAACACC |

**Supplementary methods**

***Bacillus megaterium transformation***

Protoplast of *Bacillus megaterium* de Bary were prepared as follows. 50 mL Luria broth (LB) medium was inoculated with 1 mL of preculture in 300 ml baffled flask and grow it at 37°C and strong shaking (250 rpm) to an OD_578nm_ of 1. Cells were separated from growth medium by centrifugation at 4°C, and resuspended 5 mL of freshly prepared SMMP, and transferred into a 15-mL sterile plastic reaction tube. 150 mL of freshly prepared and filter-sterilized SMMP-lysozyme solution was added to the cells and incubated at 37°C and soft shaking until 80–90% of the cells were protoplasted. Protoplasted cells were harvested carefully for 10 min and 1300 g at room temperature and the supernatant removed of the precipitated cells. Protoplasts were resuspended in 5 mL of SMMP by pipetting and spun down again. Cells were resuspended in 5 ml of SMMP and 750 ml of 87% (w/v) sterile glycerol was added. Protoplasts were stored at 80°C until use.

Protoplasts (500 µL) defrosted on ice were mixed with 10-20 µL of pure plasmid DNA (150 ng/mL) in a sterile reaction cup. The DNA–cell mixture was transferred into a 15 mL sterile tube containing 1.5 mL of PEG-P and the mixture incubated for 2 min at room temperature, after which 5 mL of freshly prepared SMMP was added and carefully mixed. The mixture was centrifuged for 10 min and 1300 g at room temperature and the supernatant discarded. The cells were re-suspended in 500 µL of SMMP and transferred into a 1.5-mL reaction tube. The cells were then incubated for 45 min without shaking followed by 45 min at 300 rpm. After the 90 min incubation time, the cell suspension was pipetted to a tube containing CR5-top-agar, mixed gently by rolling the tube, and the top agar containing the cells was poured on pre-warmed (30°C) LB medium agar plates containing the Tetracycline. The plates were incubated for up to 24 h at 30°C, and grown colonies were separated into single colonies. One of the clones were selected and used in this study.

***Culture conditions, protein preparation and purification***

BL21 cells containing pET28A^+^-P450 were grown (37°C, 220 rpm) overnight in 5 mL LB medium in the presence of kanamycin (30 µg/mL). 500 mL of Terrific Broth (TB) culture medium was started the next day with 1 mL of overnight culture until OD_600_ reached 0.8. Temperature was then lowered to 28°C and agitation to 180 rpm. Isopropyl ß-D-1-thiogalactopyranoside (IPTG) (1 mM), δ-aminolevulinic acid (1 mM), thiamin (1 mM) and iron(II) sulfate (FeSO_4_, 1 mM) were added to the culture. After 18 hours, cells were collected by centrifugation (8,000 g for 30 min). Bacterial pellets were resuspended in phosphate buffer (0.1 M) containing protease inhibitor cocktail (cComplete Millipore Sigma), DNAse I (0.5 unit/mL, Millipore Sigma) and Lysozyme (0.5 mg/mL, Millipore Sigma) and then sonicated (Sonic Dismembrator 550 and CL4 convertor probe from Fisher Scientific, Saint-Laurent, Quebec Canada) 4 × 20 seconds on ice. Cells were then centrifuged at 10,000 g for 30 min and supernatant was collected and again centrifuged at 100,000 g for one hour. 10 mM of imidazole was added to the 100,000 g supernatant and then loaded onto a column containing Ni-NTA equilibrated with Lysis buffer (50 mM NaH_2_PO_4_, 300 mM NaCl, 10 mM imidazole pH 8). Agarose was washed with wash buffer (containing 20 mM imidazole pH 8) and proteins were eluted with elution buffer (300 mM imidazole) and collected. After overnight dialysis in 0.1 M phosphate buffer pH 7.4 to remove imidazole, proteins were assayed.

***Chlamydomonas reinhardtii* transformation**

The CC-4388 strain was grown in 100 mL of liquid TAP media to mid-log growth phase (~3 days) until they reached a density of 5 × 10^6^ cells/mL. The cells were harvested by centrifuging at 3000 rpm for 3 mins and the supernatant was dissolved in 1.2 mL of HSM and 300 uL was put into 5, 13 mL sterile glass tubes with 0.3 g glass beads (0.5 mm) to generate approximately 1 × 10^8^ cells per tube. We then introduced10 µg of linearized transforming DNA (either pASapIP450BM3-1 or the empty pASapI vector as a control), and 100 µL 20% (w/v) PEG-8000 (Fisher Scientific) to a 13 mL glass tube. Cells were vortexed for 25 sec at the top setting of a Vortex-Genie mixer, washed twice with 10 mL HSM, resuspended in 300 µL HSM, mixed with 1.5 mL 0.5% Top Agar in HSM (melted at 45°C), and poured onto HSM plates, where the non-transformant cells cannot survive. The plates were incubated at 23°C at low light (~20 µmol photons m^-2^ s^-1^) overnight then transferred to a medium light (⁓50 µmol photons m^-2^ s^-1^) the next day. Transformant colonies were picked after approximately 2 weeks and re-streaked from single colonies three times on selective medium to ensure homoplasmicity.
